# Mendelian Randomization Study of Heart Failure and Stroke Subtypes

**DOI:** 10.3389/fcvm.2022.844733

**Published:** 2022-04-07

**Authors:** Quan Li, Shijiao Yan, Yan Li, Hai Kang, Huadong Zhu, Chuanzhu Lv

**Affiliations:** ^1^Emergency Department, State Key Laboratory of Complex Severe and Rare Diseases, Peking Union Medical College Hospital, Chinese Academy of Medical Science and Peking Union Medical College, Beijing, China; ^2^Emergency Medicine Center, Sichuan Provincial People's Hospital, University of Electronic Science and Technology of China, Chengdu, China; ^3^School of Public Health, Hainan Medical University, Haikou, China; ^4^Research Unit of Island Emergency Medicine, Chinese Academy of Medical Sciences (No. 2019RU013), Hainan Medical University, Haikou, China; ^5^Department of Emergency, Affiliated Yantai Yuhuangding Hospital of Qingdao University, Yantai, China; ^6^Key Laboratory of Emergency and Trauma of Ministry of Education, Hainan Medical University, Haikou, China

**Keywords:** heart failure, intracerebral hemorrhage, ischemic stroke, Mendelian randomization, stroke

## Abstract

**Background:**

Whether heart failure (HF) is an independent risk factor of ischemic stroke (IS) and hemorrhagic stroke remains controversial. We employed a multivariable Mendelian randomization (MR) to further investigate the causal effects of HF on the risk of stroke and stroke subtypes.

**Methods:**

Genetically predicted HF was selected as an instrumental variable (IV) from published genome-wide association studies (GWAS) meta-analyses. Stroke data with different etiologies were extracted as outcome variables from another two GWAS meta-analyses. The random-effects inverse variance-weighted (IVW) model was applied as the main method, along with sensitivity analysis. Atrial fibrillation (AF), coronary heart disease (CHD), and systolic blood pressure (SBP) were controlled for mediating effects in multivariable MR.

**Results:**

Genetically predicted HF was significantly associated with any IS [odds ratio (OR), 1.39; 95% CI, 1.12–1.74; *p* = 0.03], large artery stroke (LAS; OR, 1.84; 95% CI, 1.27–2.65; *p* = 0.001), and cardioembolic stroke (CES; OR, 1.73; 95% CI, 1.21–2.47; *p* = 0.003), but without small vessel stroke (SVS; OR, 1.1; 95% CI, 0.80–1.52; *p* = 0.56) and intracerebral hemorrhage (ICH; OR, 0.86; 95% CI, 0.41–1.83; *p* = 0.699) in univariable MR. However, these significant associations were attenuated to the null after adjusting for confounding factor in multivariable MR.

**Conclusion:**

There was no direct causal association between HF and stroke in our study. The association between HF and IS can be driven by AF, CHD, and SBP.

## Introduction

Stroke is now the leading cause of global mortality and disability, with an increasing global burden (1). Ischemic stroke (IS) is the major cause of the etiopathogenetic burden of a cerebrovascular accident, accounting for up to 80% of cases, with the remaining 20% being hemorrhagic strokes (2). With the growing burden of stroke, novel prevention strategies that target modifiable risk factors are urgently needed. Cardiovascular disease risk factors, such as atrial fibrillation (AF), coronary artery atherosclerosis, and hypertension, are well-known as independent risk factors for stroke (2–4). Recently, some observational studies reported that heart failure (HF) was also an independent risk factor for both IS and hemorrhagic stroke, but the evidence is controversial (3, 5–7). Zhang et al. supported that HF has a causal effect on IS (7) but was denied by Frerich et al. (3). Alberts et al. observed that HF was associated with decreased risk of hemorrhagic stroke (6), but Kasper and Danish et al. reported converse results (5, 8). Obviously, evidence regarding the causal association between HF and stroke and its subtypes is scant.

Evidence from observational studies is limited by residual confounding and detection bias (9). Stroke and HF are heterogeneous diseases involving different pathophysiological mechanisms (10, 11), and evidence for their association remains challenging based on observational studies alone. In the absence of large-scale randomized clinical trials (RCTs), a Mendelian randomization (MR) study is a novel strategy for estimating the causal effects of risk factors (12, 13). MR takes advantage of the instrumental variable (IV) method to explore the causality between a risk factor and a disease (12). MR using genetic variation as IVs mimics the design of RCTs due to the random allocation of genotypes at conception are thus able to diminish bias from confounding factors compared to observational studies (14). HF has been reported to increase the risk of IS, which may be associated with thromboembolic complications and increased activity of procoagulant factors (15, 16). However, evidence regarding the causal effects of HF on diverse etiologies of stroke [intracerebral hemorrhage (ICH), large artery stroke (LAS), small vessel stroke (SVS), and cardioembolic stroke (CES)] (17) remains a subject of debate. Herein, by leveraging large samples of genetic data on HF (18) and stroke (19), we conducted a multivariable MR analysis to examine the direct effects of HF on stroke and stroke subtypes.

## Methods

### Study Design

**Figure 1:**
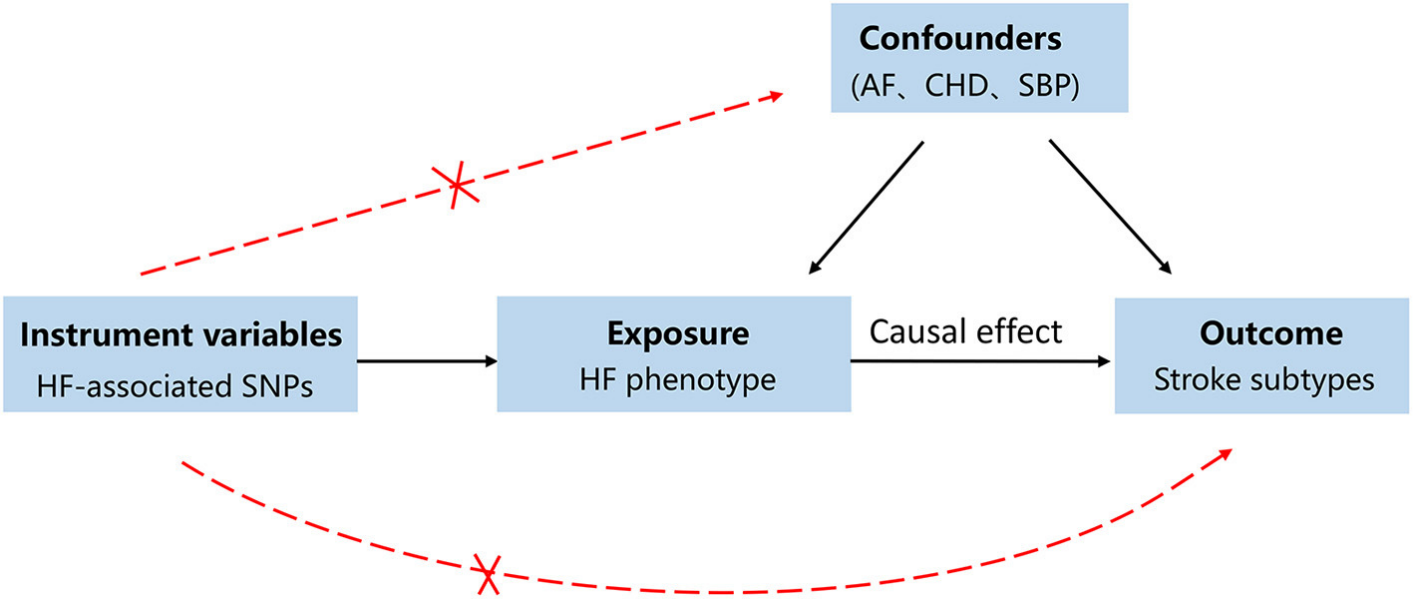
A directed acyclic graph for the Mendelian randomization study design. We selected single nucleotide polymorphisms (SNPs) associated with heart failure (HF) as genetic instruments to estimate if the HF causally influences the stroke. To make causal inference for the effect of HF on stroke, we must additionally assume that the selected SNPs is not to be associated with confounders or the outcome. In order to address the bias caused by confounders, atrial fibrillation (AF), coronary heart disease (CHD), and systolic pressure (SBP) were included as confounding factors.

A directed acyclic graph was used to assess the causal effect between HF and stroke subtypes (Figure 1). Single-nucleotide polymorphisms (SNPs) directly associated with HF were selected as IVs in the present study (18). To investigate the underlying pathogenesis linking HF to stroke and thus the subtypes of stroke were also included in the present study. We performed a two-sample MR as a univariable analysis to estimate the effect of HF on the risk of stroke and stroke subtypes. Univariable MR cannot control statistical bias due to the effect of multiple risk variables. Thus, we performed multivariable MR to estimate the direct association between HF and stroke by adjusting for putative cardiovascular risk factors, such as AF, coronary heart disease (CHD), a proxy for atherosclerosis, and systolic blood pressure (SBP). Proxy SNPs obtained from European population genotype data from the 1,000 Genomes Project (linkage disequilibrium threshold *r*^2^ ≥ 0.8) were used for those unavailable in the stroke database (20). The palindromic SNPs were directly excluded from the subsequent MR analysis to ensure the accuracy of the results. To further assess the role of heterogeneity and pleiotropic effects, we performed sensitivity analyses, which are shown in the Supplementary Material.

### Data Sources

We extracted the SNPs associated with HF at genome-wide significance (*p* < 5 × 10^−8^) with the stringent pairwise linkage disequilibrium *R*^2^ < 0.001 from a published genome-wide association studies (GWAS) meta-analysis. This GWAS meta-analysis pooled 26 studies, i.e., 47,309 HF patients and 930,014 controls from the Heart Failure Molecular Epidemiology for Therapeutic Targets Consortium (18). The analytic cohort was restricted to individuals of European descent to control for confounding by ancestry. The mean age of cases was 71.4 years and controls were 52.4 years old. The definition of HF was based on self-reported HF/pulmonary edema or international classification of disease codes for HF. The GWAS was adjusted for sex, age, and principal components. A more detailed description of GWAS has been published previously (18). The genetic data for IS and subtypes were extracted from a large GWAS meta-analysis that includes 29 studies with 40,585 cases and 406,111 controls released by the MEGASTROKE consortium (19). We restricted the stroke population to European ancestry to minimize population stratification bias. A total of 34,217 IS cases were included in the present study. The subtype of IS was classified according to the Trial of Org comprising 10,172 patients in Acute Stroke Treatment criteria (21). The subtypes were comprised of CES (*n* = 7,193), SVS (*n* = 5,386), and LAS (*n* = 4,373). IS was defined as a rapidly developing sign of focal (or global) neurological deficit enduring at least 24 h or until death with no obvious cause other than that of vascular origin (22). The genetic data for ICH were extracted from a famous GWAS meta-analysis released by the International Stroke Genetics Consortium, comprising 1,545 ICH patients and 1,481 controls of European ancestry (23). ICH cases were characterized by new and acute (<24 h) neurological deficits with consistent findings on brain imaging (24). Patients were restricted to spontaneous ICH, and those with secondary ICH caused by any other cause were excluded.

As with the primary instruments, genetic data of confounding factors, such as AF, CHD and SBP, were extracted from summary-level data from similar populations of predominantly European ancestry. AF data were extracted from six contributing studies [UK Biobank, the AFGen Consortium, deCODE, the Nord-Trøndelag Health Study (HUNT), and the Michigan Genomics Initiative (MGI), DiscovEHR] with a total of 60,620 cases and 970,216 controls of European ancestry (25). The diagnosis of AF was identified by ICD-9 and ICD-10 codes. The age at first AF diagnosis ranged from 50 to 83 years old. CHD data were from UK Biobank (34,541 CAD cases and 261,984 controls) with the mean age of 63.4 years old and CARDIoGRAMplusC4D (8,192 cases and 162,544 controls) (26). SBP data included a total of 757,601 individuals of European ancestry from UK Biobank (*N* = 458,577) and the International Consortium of Blood Pressure (*N* = 299,024) database. The mean age of participants ranged from 56.8 to 62.1 years old with a mean SBP of 138.4 mm Hg (27). All data on the included studies are available in Table 1.

**Table 1:** Descriptive characteristics of the GWAS meta-analyses used in the current Mendelian randomization study.

**Phenotype**	**Source**	**Cases**	**Control**	**Ancestry**	**Adjustments^a^**
Heart failure	HERMES	47,309	930,014	European	Age, sex
AF	UKB, the AFGen Consortium et al.	60,620	970,216	European	Age, sex
CHD	CARDioGRAMplusC4D and UKB	122,733	424,528	European (>91%)	Age, sex, age^2^, BMI
SBP	International Consortium of Blood Pressure and UKB	—	757,601	European	Age, sex
Ischemic stroke	MEGASTROKE	34,217	406,111	European	Age, sex
LAS	MEGASTROKE	4,373	406,111	European	Age, sex
CES	MEGASTROKE	7,193	406,111	European	Age, sex
SVS	MEGASTROKE	5,386	192,662	European	Age, sex
ICH	ISGC	1,545	1,481	European	Age, sex

*AF, atrial fibrillation; BMI, body mass index; CES, cardioembolic stroke; CHD, coronary heart disease; ICH, intracerebral hemorrhage; ISGC, International Stroke Genetics Consortium; GWAS, genome-wide association studies; LAS, large artery stroke; SVS, small vessel stroke; SBP, systolic blood pressure; UKB, UK Biobank*.

a *All GWAS studies have further adjusted for principal components*.

### Statistical Analysis

For univariable MR analysis, the random-effects inverse variance-weighted (IVW) meta-analysis was conducted as the primary analysis to estimate the associations between HF and stroke subtypes (28). The MR pleiotropy residual sum and outlier (MR-PRESSO) method was applied to examine pleiotropic outlier SNPs (29). Significant outlier SNPs (*p* < 0.05) were removed in the subsequent MR analysis. The IVW method is equivalent to a weighted regression of SNP-outcome effects on SNP exposure, with the y-axis intercept fixed to zero (30). Considering that the potential horizontal pleiotropy may cause bias in association statistics, we performed a sensitivity analysis with alternative MR models regarded as more robust to pleiotropic SNPs, but at the cost of reduced statistical power. In the first sensitivity analysis, we used the weighted median method, which can generate valid estimates under the condition of at least 50% of the information contributing to the analysis comes from valid SNPs (31). In the second sensitivity analysis, we also performed the MR-Egger regression, which can provide a valid estimate of the causal effect and depends on the assumption that the pleiotropic effects of genetic instruments are independent of instrument strength (32). The intercept from MR-Egger regression tested with a *p* < 0.05 is an indication of average directional pleiotropy (33). Generally, the MR-Egger method is considered conservative in the presence of pleiotropic variants and suffers from less precise estimates (31). A genome-wide suggestive significance (*p* < 5^e−6^) with more IVs was provided as a sensitivity analysis to estimate the direct causal association between HF and stroke. Statistically significant findings (*p* < 0.05) of univariable MR analysis were further investigated in subsequent multivariable MR analysis by adjusting for AF, CHD, and SBP. For multivariable MR, the multiplicative random-effects IVW method was performed as the primary analysis method, a Bonferroni correction threshold (*p* < 0.05/X/Y, X means the number of exposure phenotypes, Y means the number of outcome phenotypes) was used to ascertain statistical significance. The strength of the genetic instrument was tested using F-statistics (34). F-statistics >10 indicated a small possibility of weak instrument bias (34). All statistical analyses were conducted using R (version 4.1.1) using the TwoSampleMR and MR-PRESSO packages (30).

## Results

**Figure 2:**
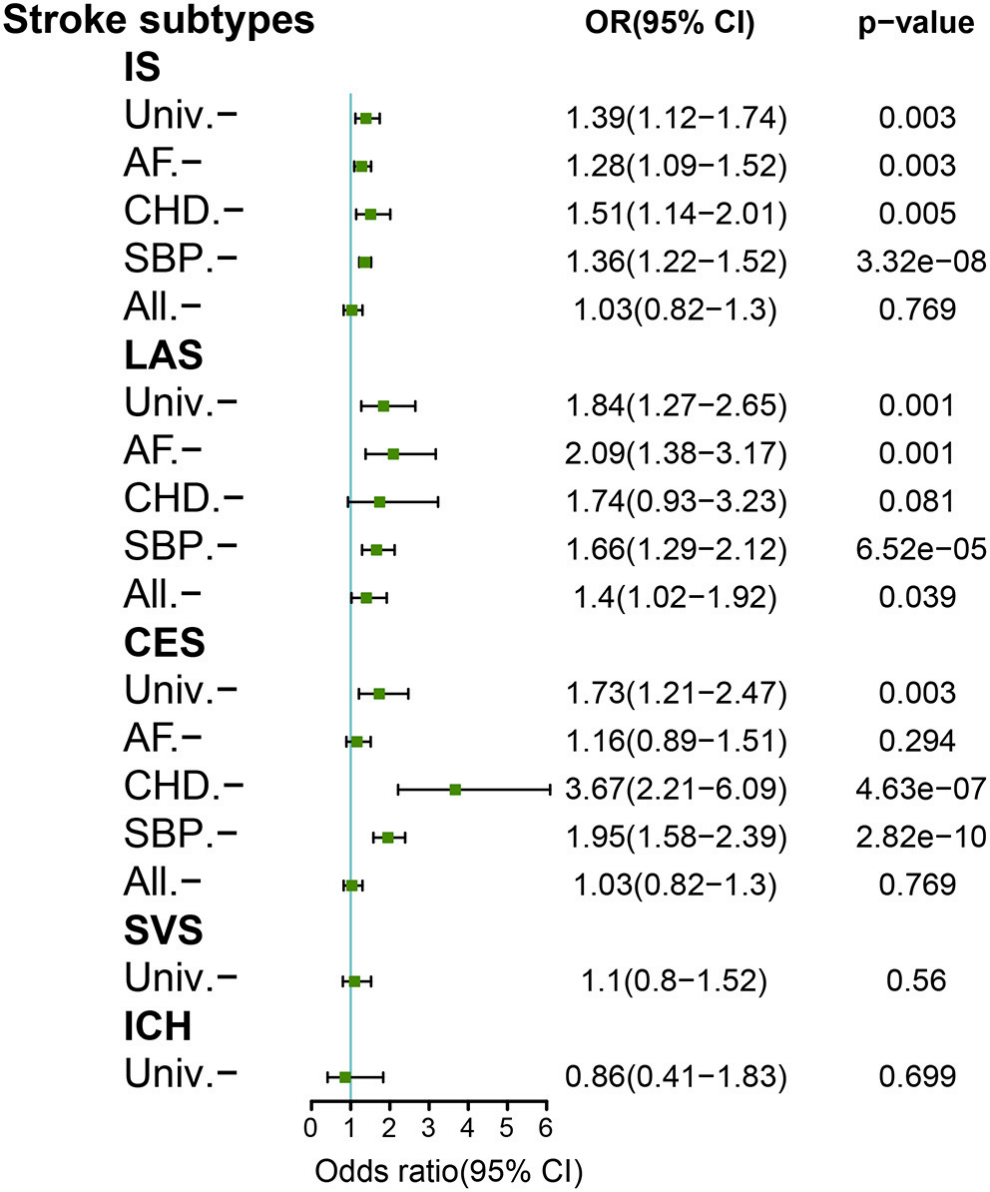
Mendelian randomization analyses of genetically predicted HF with stroke subtypes. Adjustment for atrial fibrillation (AF), coronary heart disease (CHD), and systolic blood pressure (SBP). All, combined adjustment for AF/CHD/SBP; IS, ischemic stroke; CES, cardioembolic stroke; ICH, intracerebral hemorrhage; LAS, large-artery stroke; SVS, small-vessel stroke; Univ, univariable.

After data harmonization for HF and stroke, 11 SNPs for HF were identified as genetic instruments to examine the causal association between HF and stroke subtypes. The harmonized SNPs and outliers are presented in Supplementary Table 1. The F-statistic for all variants was much >10 in the current study, indicating that the possibility of weak instrument bias was reasonably small. In the primary random-effects IVW of univariable MR analysis, genetically predicted HF was significantly associated with an increased risk of any IS [odds ratio (OR), 1.39; 95% CI, 1.12–1.74; *p* = 0.03], LAS (OR, 1.84; 95% CI, 1.27–2.65; *p* = 0.001), and CES (OR, 1.73; 95% CI, 1.21–2.47; *p* = 0.003). We found no causal association of genetically predicted HF with SVS (OR, 1.1; 95% CI, 0.80–1.52; *p* = 0.56) or ICH (OR, 0.86; 95% CI, 0.41–1.83; *p* = 0.699). However, the causal association between HF and LAS, CES and any IS was no longer statistically significant after adjusting for CHD, AF, and all of the three confounders, respectively, in a multivariable MR analysis (LAS, OR, 1.74; 95% CI, 0.93–3.23; *p* = 0.081; CES, OR 1.16; 95% CI, 0.89–1.51; *p* = 0.294; IS OR, 1.03; 95% CI, 0.82–1.3; *p* = 0.769). Significant heterogeneity was shown in the association of HF with any IS, LAS, and CES from the MR-PRESSO global test (Supplementary Table 2). There was no evidence of horizontal pleiotropic effects for stroke in the MR Egger Intercept test (Supplementary Table 2). In the univariable MR analysis, the results of sensitivity analyses (MR Egger, weighted median, simple mode, and weighted mode) showed consistency on the analysis of SVS and ICH subtypes, but inconsistency on any IS, LAS, and CES subtypes (Supplementary Figure 1). A genome-wide suggestive significance (*p* < 5^e−6^; *n* = 52) as a sensitivity analysis validated the consistent results (Supplementary Figure 2). The effect of genetically predicted HF on stroke and stroke subtypes is shown in Figure 2.

## Discussion

By leveraging large GWAS summary data in MR analyses, our study confirmed an association between HF and any IS, LAS, and CES, but no evidence of a causal association between HF and SVS or ICH. However, these associations were no longer significant after being adjusted for AF, CAD, and SBP in a multivariable MR, reiterating our findings of no direct causal effect of HF on IS or ICH, and suggesting the mediating effects driving the associations. Our multivariable MR results were robust with a genome-wide suggestive significance (*p* < 5^e−6^) as the sensitivity analyses. As expected, our study confirmed that the effect of HF on CES depended on AF, the effect of HF on LAS depended on CHD, a proxy for systemic atherosclerosis, the effect of HF on any IS depended on a comprehensive risk factor involving AF, CAD, and SBP. However, some previous observational studies regarding HF as an independent risk factor on IS remain controversial. Given that, stroke and HF are heterogeneous diseases and share many common cardiovascular risk factors, which may be easy to bias the results in observational studies. Inconsistency with our findings, a recent MR study found that HF was an independent risk factor of any IS after adjusting for a single cardiovascular risk factor (7). However, we found no causal effect of HF on any IS after adjusting for a combination of AF, CHD, and SBP. HF predisposes patients to IS in a multifactorial manner involving complex pathogenesis, and thus it may be more reasonable to adjust for comprehensive confounding factors to explore the correlation between HF and stroke.

The evidence regarding the association between HF and hemorrhagic stroke is conflicted in observational studies, with two studies reporting an increased risk of ICH (5, 8) and one showing a converse result (6). At the genetic level, we found no statistically significant causal association between HF and ICH. The pathophysiological pathway that links HF and ICH is less clear, inferring that the higher use of antithrombotic drugs in patients with HF may bias the results in observational studies. In addition, our study also highlights that the association between HF and stroke subtypes that depends on multiple risk factors, and thus the prevention strategy for stroke should be individualized. Our study benefited from methodological strength by using MR analysis, which can reduce confounding due to the random allocation of genotypes from parents to offspring, thereby minimizing the possibility of bias. Moreover, our MR analysis provided sufficient statistical power to examine the effects of HF on diverse stroke subtypes by taking advantage of a large sample size. Our MR study provided no support for a direct causal association between HF and stroke and its subtypes, which is in line with Frerich et al. (3).

Based on the current clinical practice, antiplatelet and anticoagulants are commonly pharmacological therapeutic strategies for stroke prevention (35). Aspirin remains a widely accepted drug for stroke prevention for HF patients with atherosclerotic disease. Oral anticoagulant drugs are widely accepted for HF patients with AF based on the (Congestive heart failure, Hypertension, Age, Diabetes mellitus, Stroke, Vascular disease, Age, Sex) CHA2-DS2-VASc score (35). What is more, amount of clinical trials on developing novel pharmacologically therapeutic strategies for stroke prevention in patients with HF are ongoing (36). Our study advanced the current knowledge that HF is an indirect risk factor for stroke. Therefore, in clinical practice, therapeutic strategies for stroke prevention should be individually adjusted by the presence of comorbidities.

### Limitations

The results of this study have some limitations. First, the results of additional MR methods (MR Egger, weighted median, simple mode, and weighted mode) were not completely in line with the IVW method in the univariable MR analysis. HF and stroke are heterogeneous diseases, and we were unable to fully rule out the possibility of pleiotropy in this situation. However, most estimates were consistent with sensitivity analysis, which indicates that the observed associations are robust and are not likely to be by chance observations. Second, we were limited by the fact that MR analyses usually evaluate the effects of life-long exposure on the outcomes and thus might be biased by the effects of clinical interventions with shorter periods of exposure. The effect sizes estimated in this study were limited to explaining the associations between critical periods of exposure and outcomes (37). Third, we lacked data on the classification and severity of HF. We were unable to separately evaluate the potential differences in stroke risk among different classifications and severities of HF and could not examine non-linear associations. Fourth, the analysis of ICH based on a small sample size may lead to insufficient power to detect the effects of HF on ICH. Future studies with larger GWAS datasets for ICH need to be conducted. Fifth, our analyses were restricted to individuals of European ancestry and might thus not be applicable to other ethnic groups. Future MR studies in other ethnicities are needed to extend our findings.

## Conclusion

In conclusion, at genetic levels, we found no evidence of a direct causal association between HF and stroke and its subtypes. Our study highlights the importance of controlling for confounding biases in exploring the genetic correlation between two heterogeneous diseases, such as HF and stroke.

## Data Availability Statement

The datasets presented in this study can be found in online repositories. The names of the repository/repositories and accession number(s) can be found at: https://gwas.mrcieu.ac.uk/.

## Author Contributions

CL and HZ conceived and designed the study. QL analyzed the data and drafted the manuscript. SY participated in the analysis of data and revised the manuscript. YL and HK prepared all the figures. All authors read and approved the final manuscript.

## Funding

This study was supported by CAMS Innovation Fund for Medical Sciences (2019-I2M-5-023) and Hainan Provincial Key Research and Development Project (ZDYF2020112).

## Conflict of Interest

The authors declare that the research was conducted in the absence of any commercial or financial relationships that could be construed as a potential conflict of interest.

## Publisher's Note

All claims expressed in this article are solely those of the authors and do not necessarily represent those of their affiliated organizations, or those of the publisher, the editors and the reviewers. Any product that may be evaluated in this article, or claim that may be made by its manufacturer, is not guaranteed or endorsed by the publisher.

## Abbreviations

AF: Atrial fibrillation
CES: Cardioembolic stroke
CHD: Coronary heart disease
CI: Confidence interval
GWAS: Genome-wide association studies
HF: Heart failure
ICH: Intracerebral hemorrhage
IV: Instrumental variable
IVW: Inverse variance-weighted
LAS: Large artery stroke
MR: Mendelian randomization
MR-PRESSO: MR pleiotropy residual sum and outlier
OR: Odds ratio
RCT: Randomized clinical trial
SBP: systolic blood pressure
SNP: Single-nucleotide polymorphism
SVS: Small vessel stroke.
