# Supplementary material for: Mendelian Randomization Study of Heart Failure and Stroke Subtypes
Source: Front Cardiovasc Med. 2022 Apr 7;9:844733. doi: 10.3389/fcvm.2022.844733 (PMC9021833; doi:10.3389/fcvm.2022.844733)
Supplement: Supplementary file 1 [file Data_Sheet_1.PDF]

SUPPLEMENTARY MATERIAL

CONTENTS LIST

Supplementary Tables

Supplementary Table 1. Characteristics of the genetic variants associated with stroke and stroke subtypes.
Supplementary Table 2. Estimating heterogeneity and pleiotropic effects.

Supplementary Figures

Supplementary Figure 1. Mendelian randomization analysis on the association between heart failure and stroke subtypes with additional methods.

Supplementary Figure 2. Mendelian randomization analyses of genetically predicted HF with stroke subtypes in a genome-wide suggestive significance (p < 5e-6).

STROBE-MR checklist

Supplementary Table 1. Characteristics of the genetic variants associated with stroke and strok subtypes

SNP	effect_allele	other_allele	beta.exposure	beta.outcome	se.outcome	outcome	se.exposure	mrpresso_pval	mrpresso_keep
rs11745324	A	G	-0.0528	-0.0203	0.0121	IS	0.0095	1	TURE
rs1510226	C	T	0.162	0.061	0.0402	IS	0.0285	1	TURE
rs1556516	C	G	0.0622	0.0482	0.0099	IS	0.0078	1	TURE
rs17042102	A	G	0.1103	0.1019	0.0152	IS	0.0121	0.033	FALSE
rs17617337	T	C	-0.0561	0.0039	0.0118	IS	0.0095	0.077	TURE
rs4135240	C	T	-0.0486	-0.0427	0.0106	IS	0.0084	1	TURE
rs4746140	C	G	-0.0666	-0.0093	0.0139	IS	0.0109	0.429	TURE
rs4766578	A	T	-0.0433	-0.071	0.01	IS	0.0079	<0.011	FALSE
rs55730499	T	C	0.1058	0.0463	0.0215	IS	0.0157	1	TURE
rs56094641	G	A	0.0454	0.0105	0.0102	IS	0.008	1	TURE
rs660240	C	T	0.0611	0.0087	0.0122	IS	0.0097	0.363	TURE
rs11745324	A	G	-0.0528	-0.017	0.0295	LAS	0.0095	1	TURE
rs1510226	C	T	0.162	0.1417	0.1004	LAS	0.0285	1	TURE
rs1556516	C	G	0.0622	0.0902	0.0242	LAS	0.0078	1	TURE
rs17042102	A	G	0.1103	0.0258	0.0378	LAS	0.0121	0.198	TURE
rs17617337	T	C	-0.0561	-0.0121	0.0298	LAS	0.0095	1	TURE
rs4135240	C	T	-0.0486	-0.0629	0.0264	LAS	0.0084	1	TURE
rs4746140	C	G	-0.0666	0.0205	0.034	LAS	0.0109	0.154	TURE
rs4766578	A	T	-0.0433	-0.099	0.0249	LAS	0.0079	0.132	TURE
rs55730499	T	C	0.1058	0.2497	0.0526	LAS	0.0157	0.022	FALSE
rs56094641	G	A	0.0454	0.0355	0.0253	LAS	0.008	1	TURE
rs660240	C	T	0.0611	0.0665	0.0304	LAS	0.0097	1	TURE
rs11745324	A	G	-0.0528	-0.0761	0.0235	CES	0.0095	1	TURE
rs1510226	C	T	0.162	0.0523	0.0834	CES	0.0285	1	TURE
rs1556516	C	G	0.0622	0.0038	0.0189	CES	0.0078	0.011	FALSE
rs17042102	A	G	0.1103	0.3473	0.0273	CES	0.0121	<0.011	FALSE
rs17617337	T	C	-0.0561	0.0532	0.0229	CES	0.0095	<0.011	FALSE
rs4135240	C	T	-0.0486	-0.0326	0.0202	CES	0.0084	1	TURE
rs4746140	C	G	-0.0666	-0.0852	0.0275	CES	0.0109	1	TURE
rs4766578	A	T	-0.0433	-0.0488	0.0191	CES	0.0079	1	TURE
rs55730499	T	C	0.1058	0.0412	0.043	CES	0.0157	1	TURE
rs56094641	G	A	0.0454	0.0153	0.0192	CES	0.008	1	TURE
rs660240	C	T	0.0611	0.0128	0.0237	CES	0.0097	0.418	TURE
rs11745324	A	G	-0.0528	-0.0382	0.0277	SVS	0.0095	NA	TURE
rs1510226	C	T	0.162	0.0655	0.0944	SVS	0.0285	NA	TURE
rs1556516	C	G	0.0622	0.041	0.0226	SVS	0.0078	NA	TURE
rs17042102	A	G	0.1103	0.0033	0.036	SVS	0.0121	NA	TURE
rs17617337	T	C	-0.0561	0.0047	0.0277	SVS	0.0095	NA	TURE
rs4135240	C	T	-0.0486	-0.0076	0.0244	SVS	0.0084	NA	TURE
rs4746140	C	G	-0.0666	0.0292	0.0321	SVS	0.0109	NA	TURE
rs4766578	A	T	-0.0433	-0.0836	0.0228	SVS	0.0079	NA	TURE
rs55730499	T	C	0.1058	-0.0699	0.0497	SVS	0.0157	NA	TURE

rs56094641	G	A	0.0454	0.0174	0.0233	SVS	0.008	NA	TURE
rs660240	C	T	0.0611	0.0174	0.029	SVS	0.0097	NA	TURE
rs11745324	A	G	-0.0528	-0.0408	0.0597	ICH	0.0095	NA	TURE
rs1510226	C	T	0.162	-0.1804	0.1944	ICH	0.0285	NA	TURE
rs1556516	C	G	0.0622	0.0627	0.0514	ICH	0.0078	NA	TURE
rs17042102	A	G	0.1103	0.0019	0.0771	ICH	0.0121	NA	TURE
rs17617337	T	C	-0.0561	0.0865	0.0623	ICH	0.0095	NA	TURE
rs3176320	C	T	-0.0486	0.0142	0.0552	ICH	0.0084	NA	TURE
rs4746140	C	G	-0.0666	0.0392	0.071	ICH	0.0109	NA	TURE
rs56094641	G	A	0.0454	-0.0072	0.052	ICH	0.008	NA	TURE
rs600038	C	T	0.0569	-0.0135	0.0621	ICH	0.0096	NA	TURE
rs653178	C	T	-0.0433	-0.0013	0.0508	ICH	0.0079	NA	TURE
rs660240	C	T	0.0611	0.0468	0.0642	ICH	0.0097	NA	TURE

CES: Cardioembolic stroke;ICH, intracerebral hemorrhage;IS:ischemic stroke;LAS: Large artery stroke;SE: standard error; SNP: single nucleotide polymorphism;SVS: Small vessel stroke.

Supplementary Table 2. Estimating heterogeneity and pleiotropic effects

outcome	exposure	N SNP	N Outliers	MR-PRESSO Global *p*	MR-Egger Intercept *p*
IS	heart failure	11	2	<0.001	9.06E-01
LAS	heart failure	11	2	3.00E-03	6.26E-01
CES	heart failure	11	3	<0.001	1.29E-01
SVS	heart failure	11	0	5.40E-02	8.18E-02
ICH	heart failure	11	0	8.21E-01	7.29E-01

CES: Cardioembolic stroke;ICH, intracerebral hemorrhage;IS:ischemic stroke;LAS: Large artery stroke;SNP: single nucleotide polymorphism; SVS: Small vessel stroke.

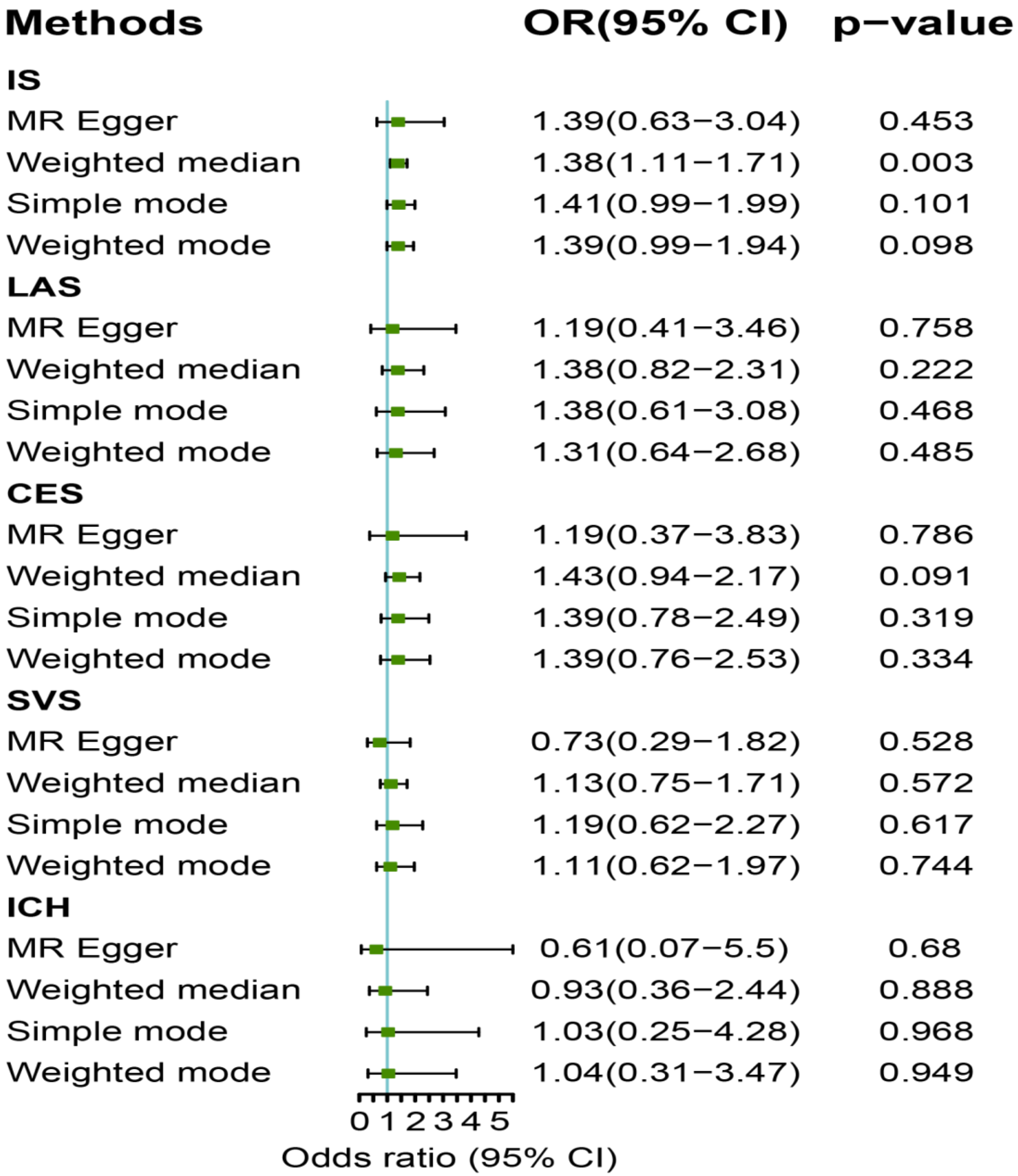

Supplementary Figure 1 Mendelian randomization analysis on the association between heart failure and stroke subtypes with additional methods. IS, ischemic stroke; CES, cardioembolic stroke; ICH, intracerebral hemorrhage; OR, odds ratio; LAS, large-artery stroke; SVS, small-vessel stroke.

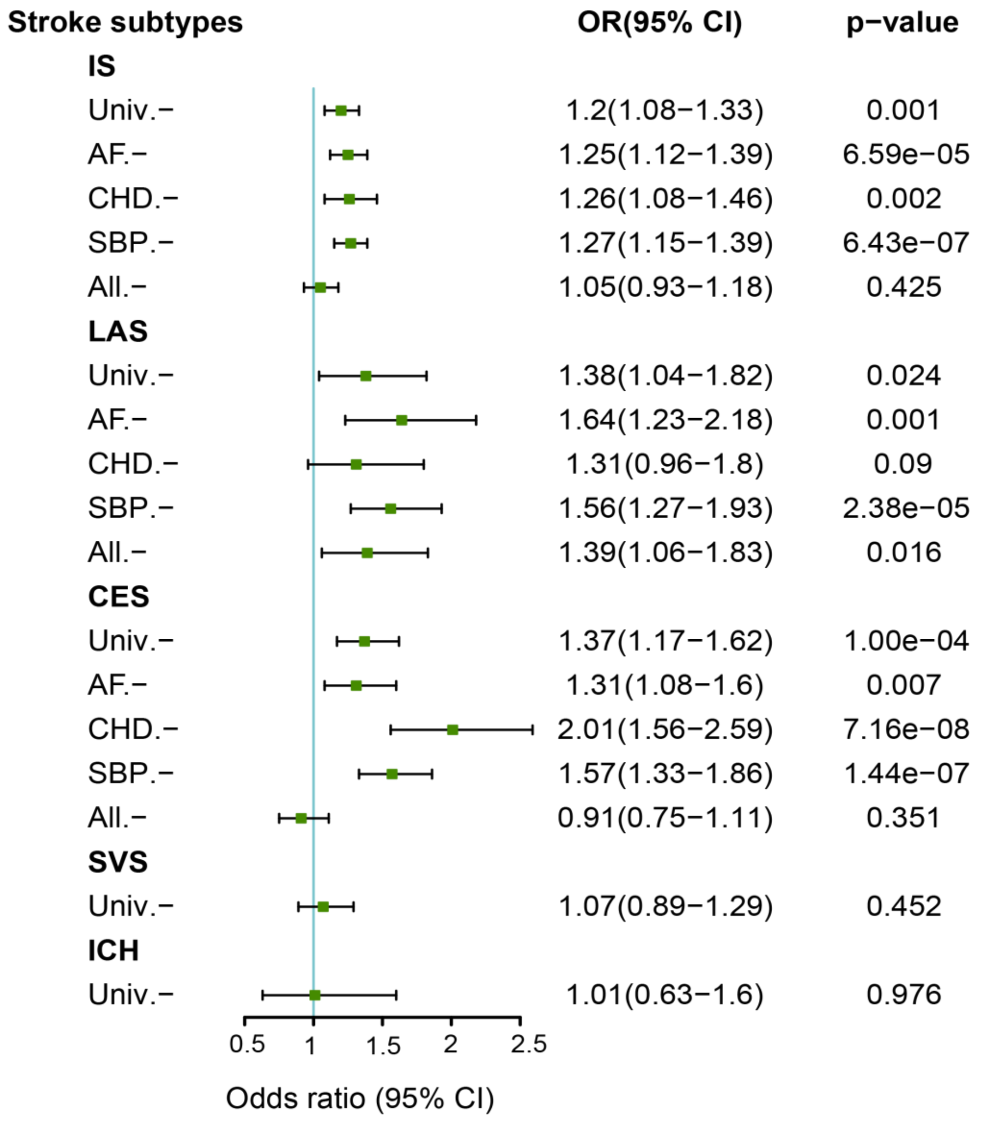

Supplementary Figure 2 Mendelian randomization analyses of genetically predicted HF with stroke subtypes in a genome-wide suggestive significance (p < 5e-6). Adjustment for atrial fbrillation (AF), coronary heart disease (CHD), and systolic blood pressure (SBP). All, combined adjustment for AF/CHD/SBP; IS, ischemic stroke; CES, cardioembolic stroke; ICH, intracerebral hemorrhage; LAS, large-artery stroke; SVS, small-vessel stroke; Univ, univariable.

STROBE-MR checklist of recommended items to address in reports of Mendelian randomization studies[1 2]

Item No.	Section		Checklist item
1	**TITLE and ABSTRACT**		Indicate Mendelian randomization (MR) as the study's design in the title and/or the abstract if that is a main purpose of the study
	**INTRODUCTION**		
2	**Background**		Explain the scientific background and rationale for the reported study. What is the exposure? Is a potential causal relationship between exposure and outcome plausible? Justify why MR is a helpful method to address the study question
3	**Objectives**		State specific objectives clearly, including pre-specified causal hypotheses (if any). State that MR is a method that, under specific assumptions, intends to estimate causal effects
	**METHODS**		
4	**Study design and data sources**		Present key elements of the study design early in the article. Consider including a table listing sources of data for all phases of the study. For each data source contributing to the analysis, describe the following:
		a)	Setting: Describe the study design and the underlying population, if possible. Describe the setting, locations, and relevant dates, including periods of recruitment, exposure, follow-up, and data collection, when available.
		b)	Participants: Give the eligibility criteria, and the sources and methods of selection of participants. Report the sample size, and whether any power or sample size calculations were carried out prior to the main analysis
		c)	Describe measurement, quality control and selection of genetic variants
		d)	For each exposure, outcome, and other relevant variables, describe methods of assessment and diagnostic criteria for diseases
		e)	Provide details of ethics committee approval and participant informed consent, if relevant
5	**Assumptions**		Explicitly state the three core IV assumptions for the main analysis (relevance, independence and exclusion restriction) as well assumptions for any additional or sensitivity analysis
6	**Statistical methods: main analysis**		Describe statistical methods and statistics used
		a)	Describe how quantitative variables were handled in the analyses (i.e., scale, units, model)
		b)	Describe how genetic variants were handled in the analyses and, if applicable, how their weights were selected
		c)	Describe the MR estimator (e.g. two-stage least squares, Wald ratio) and related statistics. Detail the included covariates and, in case of two-sample MR, whether the same covariate set was used for adjustment in the two samples
		d)	Explain how missing data were addressed
		e)	If applicable, indicate how multiple testing was addressed

7	**Assessment of assumptions**		Describe any methods or prior knowledge used to assess the assumptions or justify their validity
8	**Sensitivity analyses and additional analyses**		Describe any sensitivity analyses or additional analyses performed (e.g. comparison of effect estimates from different approaches, independent replication, bias analytic techniques, validation of instruments, simulations)
9	**Software and pre-registration**		
		a)	Name statistical software and package(s), including version and settings used
		b)	State whether the study protocol and details were pre-registered (as well as when and where)
	**RESULTS**		
10	**Descriptive data**		
		a)	Report the numbers of individuals at each stage of included studies and reasons for exclusion. Consider use of a flow diagram
		b)	Report summary statistics for phenotypic exposure(s), outcome(s), and other relevant variables (e.g. means, SDs, proportions)
		c)	If the data sources include meta-analyses of previous studies, provide the assessments of heterogeneity across these studies
		d)	For two-sample MR:<br>i. Provide justification of the similarity of the genetic variant-exposure associations between the exposure and outcome samples<br>ii. Provide information on the number of individuals who overlap between the exposure and outcome studies
11	**Main results**		
		a)	Report the associations between genetic variant and exposure, and between genetic variant and outcome, preferably on an interpretable scale
		b)	Report MR estimates of the relationship between exposure and outcome, and the measures of uncertainty from the MR analysis, on an interpretable scale, such as odds ratio or relative risk per SD difference
		c)	If relevant, consider translating estimates of relative risk into absolute risk for a meaningful time period
		d)	Consider plots to visualize results (e.g. forest plot, scatterplot of associations between genetic variants and outcome versus between genetic variants and exposure)
12	**Assessment of assumptions**		
		a)	Report the assessment of the validity of the assumptions
		b)	Report any additional statistics (e.g., assessments of heterogeneity across genetic variants, such as I^2, Q statistic or E-value)
13	**Sensitivity analyses and additional analyses**		

		a)	Report any sensitivity analyses to assess the robustness of the main results to violations of the assumptions
		b)	Report results from other sensitivity analyses or additional analyses
		c)	Report any assessment of direction of causal relationship (e.g., bidirectional MR)
		d)	When relevant, report and compare with estimates from non-MR analyses
		e)	Consider additional plots to visualize results (e.g., leave-one-out analyses)
	**DISCUSSION**		
14	**Key results**		Summarize key results with reference to study objectives
15	**Limitations**		Discuss limitations of the study, taking into account the validity of the IV assumptions, other sources of potential bias, and imprecision. Discuss both direction and magnitude of any potential bias and any efforts to address them
16	**Interpretation**		
		a)	Meaning: Give a cautious overall interpretation of results in the context of their limitations and in comparison with other studies
		b)	Mechanism: Discuss underlying biological mechanisms that could drive a potential causal relationship between the investigated exposure and the outcome, and whether the gene-environment equivalence assumption is reasonable. Use causal language carefully, clarifying that IV estimates may provide causal effects only under certain assumptions
		c)	Clinical relevance: Discuss whether the results have clinical or public policy relevance, and to what extent they inform effect sizes of possible interventions
17	**Generalizability**		Discuss the generalizability of the study results (a) to other populations, (b) across other exposure periods/timings, and (c) across other levels of exposure
	**OTHER INFORMATION**		
18	**Funding**		Describe sources of funding and the role of funders in the present study and, if applicable, sources of funding for the databases and original study or studies on which the present study is based
19	**Data and data sharing**		Provide the data used to perform all analyses or report where and how the data can be accessed, and reference these sources in the article. Provide the statistical code needed to reproduce the results in the article, or report whether the code is publicly accessible and if so, where
20	**Conflicts of Interest**		All authors should declare all potential conflicts of interest

This checklist is copyrighted by the Equator Network under the Creative Commons Attribution 3.0 Unported (CC BY 3.0) license.

1. Skrivankova VW, Richmond RC, Woolf BAR, Yarmolinsky J, Davies NM, Swanson SA, et al. Strengthening the Reporting of Observational Studies in Epidemiology using Mendelian Randomization (STROBE-MR) Statement. JAMA. 2021;under review.

2. Skrivankova VW, Richmond RC, Woolf BAR, Davies NM, Swanson SA, VanderWeele TJ, et al. Strengthening the Reporting of Observational Studies in Epidemiology using Mendelian Randomisation (STROBE-MR): Explanation and Elaboration. BMJ. 2021;375:n2233.
